# Longshengzhi Capsules Improve Ischemic Stroke Outcomes and Reperfusion Injury via the Promotion of Anti-Inflammatory and Neuroprotective Effects in MCAO/R Rats

**DOI:** 10.1155/2020/9654175

**Published:** 2020-03-09

**Authors:** Weinan Yang, Lincheng Zhang, Simiao Chen, Qigu Yao, Haihong Chen, Jing Zhou, Weiyan Chen, Lan He, Yuyan Zhang

**Affiliations:** ^1^Second Clinical Medical College, Zhejiang Chinese Medical University, Hangzhou, Zhejiang 310053, China; ^2^School of Life Science, Zhejiang Chinese Medical University, Hangzhou, Zhejiang 310053, China; ^3^Hangzhou Hospital of Traditional Chinese Medicine, Hangzhou, Zhejiang, China; ^4^Basic Medical College, Zhejiang Chinese Medical University, Hangzhou, Zhejiang 310053, China

## Abstract

Stroke is the leading cause of death in the elderly. Traditional Chinese medicine provides an exciting strategy for treating stroke. Previous reports indicated that Longshengzhi capsules (LSZ), a modified Chinese formula, reduced formed thrombi and oxidative stress and were promising in the clinical treatment of ischemic stroke. However, the specific therapeutic effect and mechanism of LSZ are still ambiguous. This study aimed to define the effects of LSZ on proinflammatory mediators and neuroprotective effects on middle cerebral artery occlusion and refusion (MCAO/R) rats. Rats were treated with different doses of LSZ (0.54, 1.62, and 4.32 g/(kg·d)) in a week after model building. LSZ could improve the survival rate, ischemic stroke outcome, and infarct volume. In addition, significant decrease was observed in reactive oxygen species (ROS) levels and inflammatory factor levels in LSZ-treated groups, concomitant with increase in activities of superoxide dismutase (SOD), neurosynaptic remodeling, and decrease in brain edema. It is proposed that LSZ has anti-inflammatory and neuroprotective effects resulting in downregulating matrix metalloproteinase 2/9 (MMP-2/9) and vascular endothelial growth factor (VEGF) and nuclear factor kappa-B (NF-*κ*B) and upregulating microtubule-associated protein-2 (Map-2) and growth-associated protein-43 (GAP-43) via p38 MAPK and HIF-1*α* signaling pathways in MCAO/R rats. This study provides potential evidences that p38 MAPK and HIF-1*α*/VEGF signaling pathways play significant roles in the anti-inflammatory and neuroprotective effects of LSZ.

## 1. Introduction

Ischemic stroke is still a leading cause of death and disability in the world, especially in ageing society [1]. Recombinant tissue plasminogen activator (r-tPA) remains the only drug approved by the Federal Drug Administration (FDA) to treat ischemic stroke by promoting thrombolysis and reopening occluded blood vessels [2]. Unfortunately, only a small percentage of patients who are suffering from ischemic stroke can be saved when treated with t-PA [3].

Following focal cerebral ischemia and refusion (I/R), the disruption of the blood-brain barrier (BBB) is aggravated via the increased expression of matrix metalloproteinases (MMPs), especially matrix metalloproteinase-2 (MMP-2) and matrix metalloproteinase-9 (MMP-9) while promoting brain edema and hemorrhage [4, 5]. Mitogen-activated protein kinases (MAPKs) are activated and serve critical roles in neuronal survival [6–8]. Accumulating evidence has established that p38 MAPK is activated in neurons, astrocytes, and microglia after various types of ischemia [9, 10]. Its prolonged activation leads to neuronal apoptosis and the production of proinflammatory cytokines, including tumor necrosis factor-*α* (TNF-*α*) and interleukin-1*β* (IL-1*β*). In turn, TNF-*α* and IL-1*β* accelerate the activation of the p38 MAPK pathway [11, 12]. Indeed, clinical trials have demonstrated that ischemic stroke was highly relevant to the levels of inflammatory markers. Activation of nuclear factor kappa-B (NF-*κ*B) is necessary for induction and production of many proinflammatory mediators, including TNF-*α* and IL-1*β* [13–15]. Furthermore, some evidence has shown that the activity of NF-*κ*B and MMPs may be induced by p38 MAPK in ischemic reperfusion injury [16, 17]. Vascular endothelial growth factor (VEGF) is increased in the hyperacute stage of cerebral ischemia. Hypoxia inducible factor-1 (HIF-1*α*) has a function in brain edema formation and BBB disruption via a signaling pathway involving VEGF, MMP-2, and MMP-9 [18]. Moreover, growth-associated protein-43 (GAP-43) and microtubule-associated protein-2 (MAP-2) are both closely related to synaptic plasticity and the neuronal cytoskeleton and are posited to play pivotal roles in neurorehabilitation [19, 20].

Traditional Chinese medicine (TCM) is characterized by the pairing of a number of herbs to enhance therapeutic effects. Compared with the administration of single herb, synergies may occur when two or more such ingredients are combined [21]. In TCM, treatments which promoted blood circulation, removed blood stasis, resolved exterior heat, and cleared away heat were used widely for ischemic stroke. Buyang Huanwu Decoction, Danhong Injection, and Qingkailing Injection are eminent Chinese herbal formulae for cerebrovascular disorders [22–24]. In this context, Longshengzhi capsules (LSZ) may be a promising choice for enhancing treatment efficacy.

LSZ was originally developed by Buchang Pharmaceutical Co. Ltd, through the modification of a prescription of traditional Chinese medicine (Buyang Huanwu Decoction). Based on the theory of TCM, LSZ can tonify the Qi, refresh blood, and unblock the meridians and collaterals. It has been approved by the China Food and Drug Administration to extensively fight cerebrovascular and cardiovascular diseases during the recovery stage of ischemic stroke in China, especially stroke with qi-deficiency and blood stasis syndrome. Clinical trials have shown that LSZ can resist arteriosclerosis and brain tissue damage as well as inflammation and even improve the prognosis of cerebral infarction and neurological function [25–29]. Previous reports indicated that the main bioactive ingredients of LSZ had neuroprotective effect and benefited in prognosis of stroke [30–34]. Additionally, other reports have shown that LSZ reduced activation of platelets and endothelial cells and inhibited systematic inflammatory reaction [35, 36]. However, there is a dearth of research on the specific therapeutic effects and the mechanism of LSZ, which has severely hindered its clinical application and development. Therefore, this study was conducted to explain the specific therapeutic effect of LSZ on stroke, as well as to conduct an initial exploration of the signaling pathway of LSZ in patients with cerebral ischemia.

**Figure 1:**
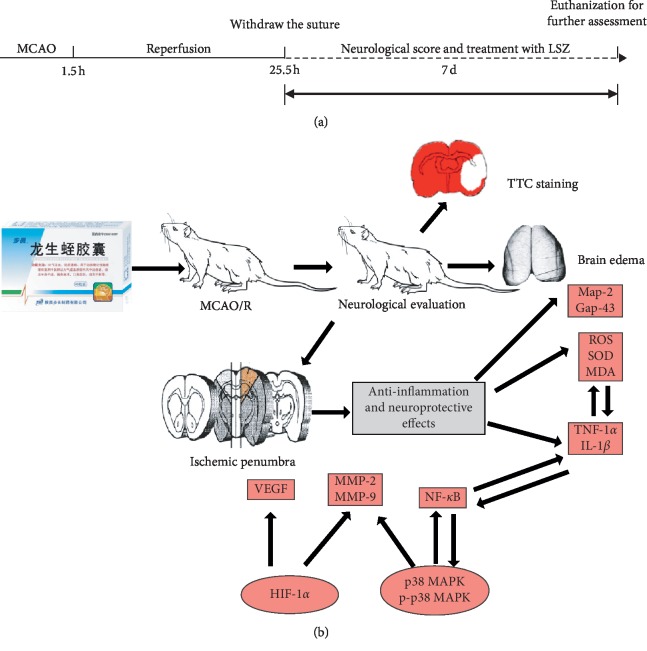
Experimental procedure and protocol (a) Treatment with LSZ (0.54, 1.62, and 4.32 g/(kg·d)) was administered via gavage after MCAO/R in rats for seven days. Following this, rats were euthanized for further assessment. (b) Neuroprotective and anti-inflammatory effects of LSZ for ischemic stroke and reperfusion injury are closely related to the HIF-1*α* and p38 MAPK signaling pathways.

It has been reported that Buyang Huanwu Decoction protected against cerebral I/R injury through inhibiting the activation of the HIF-1*α*/VEGF pathway and inflammation [37, 38]. Similarly, it has been hypothesized that p38 MAPK pathways and HIF-1*α* could be therapeutic targets of LSZ for cerebral I/R injury. This study was, therefore, conducted to define the effects of LSZ on the production of proinflammatory mediators and neuroprotective effects in the ischemic penumbra of the cerebral cortex following middle cerebral artery occlusion and refusion (MCAO/R) injury in rats (see Figure 1).

## 2. Methods

### 2.1. Quality Control of Longshengzhi Capsules

Longshengzhi capsules, approved by the China Food and Drug Administration (CFDA) (Z20010059), are manufactured by Buchang Pharmaceutical Co. Ltd. in Xianyang City, Shaanxi Province, China. Latin name, family, local name, English name, and medicinal parts of LSZ ingredients are listed in Table 1. As a capsule, a vastly strict quality control system was executed in the factory, including identification, inspection, determination and fingerprint. The moisture content is within 9%, the loading difference is ±10%, the disintegration time is within 30 minutes, the chromium content is within two parts per million, and the microbial limit meets the requirements. Consistent with existing studies, it is confirmed that the main component of LSZ (Astragaloside IV) maintains stable by HPLC and TLC [39, 40]. Moreover, a study demonstrates that other active components of LSZ also remain stable by HPLC [41]. Obeying to the corresponding quality control standard, the contents of Astragaloside IV (C_41_H_68_O_14_), Ferulic acid (C_10_H_10_O_4_) and Hydroxy safflower pigment A (C_27_H_32_O_16_) determined by HPLC are not less than, respectively, 140 *μ*g/g, 10 *μ*g/g and 30 *μ*g/g. Currently, the qualified LSZ has been extensively used in the clinical treatment of ischemic stroke.

### 2.2. Experimental Rats and MCAO/R Model

All experiments were performed obeying protocols in accordance with the National Institute of Health Guide for the Care and Use of Laboratory Animals as well as the ARRIVE guidelines. Adult male Sprague-Dawley rats (body weight, 250–280 g) were provided by the Animal Center of Zhejiang Chinese Medical University, Hangzhou, China (Laboratory Animal Certificate: SCXK: 2014-0001). The process for making MCAO/R models has been stated in a previous paper [24]. Briefly, all rats were anesthetized with an intraperitoneal injection of 1% pentobarbital sodium (40 mg/kg for the first injection, 10 mg/kg for maintenance), and then exposed the common carotid artery (CCA), internal carotid artery (ICA), and external carotid artery (ECA) with careful blunt dissection. The CCA and the ICA were clamped with an aneurysm clip, and a 4-0 monofilament nylon suture was applied loosely around the trunk of the ECA near the bifurcation. A partial arteriotomy on the ECA is created, and then the tip of a 0.36 mm diameter nylon filament (2636A2, Beijing Cinontech Biotech Co. Ltd., Beijing, China) was inserted into the arteriotomy. After 1.5 hours, the intracarotid nylon filament was removed for refusion. Moreover, all rats were allowed to freely access food and water, but fasting for 12 hours before surgery. Inclusion criteria were that rats had to have not more than 50% regional cerebral blood flow of the preischemic baseline for up to 1 hour after embolization [42].

### 2.3. Experiments and Treatment Groups

Rats were randomized into six groups: sham group, MCAO/R group, LSZ groups, and Nimodipine group. In LSZ groups, rats were treated with LSZ (lot no.170618, Buchang Pharmaceutical Co. Ltd., Xianyang City, Shaanxi Province, China) at doses of 0.54, 1.62, and 4.32 g/(kg·d). In the Nimodipine group, rats were treated with Nimodipine (Bayer, Leverkusen, Germany) at a dose of 0.01 g/(kg·d). The equivalent dose was calculated according to the body surface area between humans and rats as well as clinical treatment dose of LSZ and Nimodipine [43, 44]. Nimodipine, a selective calcium channel blocker, can pass through the BBB without any obstacles and is confirmed to have neuroprotective effect on MCAO/R [45]. The final concentration of Nimodipine was identified as 2.5 mg/ml with 0.5% CMC-Na. Sham group, MCAO/R group, LSZ groups and Nimodipine group were administered 0.9% normal saline, LSZ, and Nimodipine by gavage at the corresponding concentration and the same volume, once a day at around 9 am for seven days. The first dose was administered 24 hours after cerebral ischemia reperfusion.

### 2.4. Evaluation of Neurological Deficit

The assessment of neurological deficits was performed after seven days of treatment by several researchers who were unaware of the groupings using a neurologic deficit score [46].

### 2.5. Measurements of Infarct Volume and Brain Edema Volume

Following the assessment of neurological deficits, rats were euthanized and their brains were rapidly removed and mildly frozen at −20°C for morphological integrity. The brain was cut into 2 mm slices from the frontal tips, among which the best six slices were selected to stain with 2% 2,3,5-triphenyltetrazolium chloride (TTC, Sigma, St. Louis, MO, United States) at 37°C for 20 minutes in the dark. The infarct part was recorded using a digital camera and quantified using image analysis.

Brain edema volume was determined through the use of the wet-dry method following neurological evaluation. Fresh brain hemispheres were weighed using an electronic scale (wet weight) and then dried until the quality did not change any more at 95°C in a desiccating oven. Dried brain hemispheres were weighed again (dry weight). Ultimately, the brain water content was determined by the following formula.

(1) $$\begin{array}{c} \text{Brain water content}=\frac{\text{Wet weight}-\text{Dry weight}}{\text{Wet weight}}\times 100\%. \end{array}$$

### 2.6. Measurements of Oxidative Damage and Inflammation Levels

The ischemic penumbra was dissected at 0°C, homogenized with cold normal saline, and centrifuged at 12,000 g for 15 minutes at 4°C, after which the supernatant was collected for ELISA detection.

In terms of oxidative damage, the antioxidant status and oxidative damage of the brain were assessed, SOD and ROS, respectively, with activity quantified using the ELISA kits (Mei-mian Biotech, Yancheng, China). The concentration of MDA, an end product of lipid peroxidation, was tested following a kit's protocol (Nanjing Jiancheng Bioengineering Institute, Nanjing, China).

The inflammation reaction level was assessed using the content of TNF-*α* and IL-1*β*, the content of which was quantified using the ELISA kits (Meimian, Jiangsu Kete Biotechnology Co., Ltd., China).

### 2.7. Reverse Transcription and Quantitative RT-PCR Analysis

Total RNA was separated from the ischemic brain using the Trizol Plus RNA Purification Kit (Thermo Fisher Scientific, Massachusetts, USA) and the RNase-Free DNase Set (Qiagen, Duesseldorf, Germany), and concentration was detected using NanoVue Plus (A260/A280). Total RNA was subsequently reverse-transcribed to cDNA using SuperScript™ III First-Strand Synthesis SuperMix for qRT-PCR (Thermo Fisher Scientific, Massachusetts, USA). The reaction condition was installed for 25°C for 10 minutes, 50°C for 30 minutes, and 85°C for five minutes and saved at −20°C. Real-time PCR was performed utilizing Power SYBR® Green PCR Master Mix (Applied Biosystems, USA). Additionally, specific primers synthesized by Sangon Biotech, Co., Ltd. (Shanghai, China) are shown in Table 2. These were then programmed to conduct one cycle at 95°C for one minute, followed by 40 cycles at 95°C for 15 seconds and 63°C for 25 seconds. GAPDH (glyceraldehyde-3-phosphate dehydrogenase) was utilized as an internal control, while data were analyzed using the comparative threshold cycle method.

### 2.8. Western Blot

In order to isolate proteins in the ischemic penumbra, samples were homogenized in lysis buffer and centrifuged at 13,000 g for 15 minutes at 4°C. Supernatants were collected and employed for protein determination using the BCA Protein Assay Kit (Beyotime Institute of Biotechnology, Nanjing, China). Samples were denatured in reducing buffer and separated on 10% SDS-PAGE. Then, proteins were transferred to polyvinylidene difluoride membranes (Millipore Corporation, Billerica, USA). Membrane was blocked with 5% nonfat dry milk in Tris-buffered saline containing 0.05% Tween-20 (TBST) buffer and then incubated using primary antibodies for HIF-1*α* (1 : 1000 dilution, Abcam, ab179483), p-p38 MAPK (1 : 1000 dilution, Cell Signaling Technology, 9211), total p38 MAPK (1 : 1000 dilution, Cell Signaling Technology, 8690), MMP-2 (1 : 500 dilution, Abcam, ab97779), and MMP-9 (1 : 1000 dilution, Abcam, ab38898) overnight at 4°C. The following day, membranes were washed three times utilizing the TBST buffer and incubated with secondary antibodies coupled with horseradish peroxidase (1 : 5000 dilution, Thermo Pierce, No.31160) for two hours at room temperature. To prove equal loading, blots were analyzed for GAPDH (housekeeping gene) expression using an anti-GAPDH antibody (1 : 10000 dilution, Abcam, ab181602). Following washing, membranes were analyzed using the enhanced chemiluminescence system, according to the manufacturer's protocol (Clinx, Shanghai, China). Protein signals were quantified with scanning densitometry using Quantity One Software (Bio-Rad Laboratories, Hercules, CA, USA). Levels of total p38 MAPK, HIF-1*α*, MMP-2, and MMP-9 were expressed as relative integrated intensity normalized vs GAPDH. The p-p38 MAPK is shown as the ratio of the integrated intensity of the phosphorylated vs the unphosphorylated form.

### 2.9. Statistical Analysis

Data were expressed as mean ± SD. Statistical analysis was performed using one-way analysis of variance (ANOVA) as well as the least significant difference test. Differences were considered statistically significant at a *P* value <0.05.

## 3. Results

### 3.1. Effect of Longshengzhi Capsules on Neurological Deficit Scores

**Figure 2:**
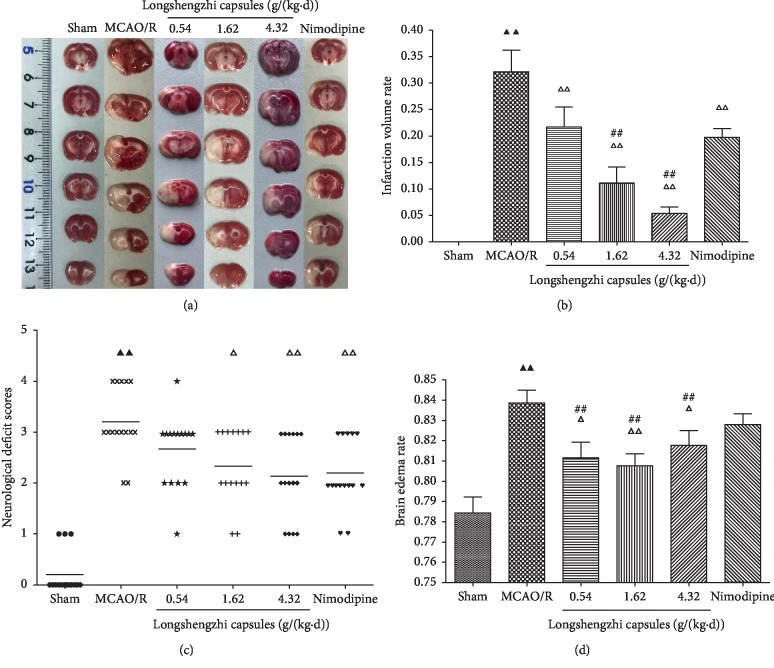
Effects of LSZ on neurological deficit scores, infarct volume, and brain edema volume. (a) Representative photographs of brain slices following infarction and stained with TTC. The red tissue is the normal part, while the white is the infarcted part. Treatment with LSZ (0.54, 1.62, and 4.32 g/(kg·d)) significantly reduced infarct volume (b), improved neurological scores (c), and decreased brain edema volume (d) when compared with the MCAO/R group. Data are expressed as mean ± SD, *n* = 6 per group. ^▲^*P* < 0.05, ^▲▲^*P* < 0.01 vs sham group. ^Δ^*P* < 0.05, ^ΔΔ^*P* < 0.01 vs MCAO/R group. ^#^*P* < 0.05, ^##^*P* < 0.01 vs Nimodipine group.

Rats subjected to MCAO/R showed significant motor behavioral deficits. In parallel with the sham group, neurological deficit scores dramatically increased in the MCAO/R group (*P* < 0.01, Figure 2(c)). Administration of both LSZ (1.62 and 4.32 g/(kg·d)) and Nimodipine (0.01 g/(kg·d)) led to a significant decrease in neurological scores when compared with the MCAO/R group (*P* < 0.01, Figure 2(c)). However, there was no statistical difference in scores between LSZ group (0.54 g/(kg·d)) and MCAO/R group, as well as LSZ (0.54, 1.62, and 4.32 g/(kg·d)) and Nimodipine (0.01 g/(kg·d)) (*P* > 0.05, Figure 2(c)).

### 3.2. Effects of Longshengzhi Capsules on Infarct Volume and Brain Edema Volume

In order to determine whether LSZ was neuroprotective, extensive infarction was visualized using TTC staining (Figure 2(a)). Rats treated with LSZ (0.54, 1.62, and 4.32 g/(kg·d)) and Nimodipine (0.01 g/(kg·d)) demonstrated significantly smaller infarct volumes than those in the MCAO/R group (*P* < 0.01, Figure 2(b)). Compared with Nimodipine (0.01 g/(kg·d)), rats treated with LSZ (1.62, 4.32 g/(kg·d)) had smaller infarct volumes (*P* < 0.01, Figure 2(b)). Meanwhile, brain edema volume was examined to assess blood-brain barrier integrity in all groups. Brain edema volume remarkably increased in the MCAO/R group and significantly reduced following LSZ treatment (0.54, 1.62, and 4.32 g/(kg·d)) (*P* < 0.01 or *P* < 0.05, Figure 2(d)). In contrast, rats which had been treated with Nimodipine (0.01 g/(kg·d)) demonstrated no difference with those in the MCAO/R group (*P* > 0.05, Figure 2(d)) and significantly higher brain edema rates than LSZ (0.54, 1.62, and 4.32 g/(kg·d)) (*P* < 0.01, Figure 2(d)). Moreover, no infarction or edema was found in the sham group (Figures 2(b) and 2(d)).

### 3.3. Effects of Longshengzhi Capsules on ROS, MDA, and SOD Levels for Treatment of Ischemic Stroke in Rats after MCAO/R

**Figure 3:**
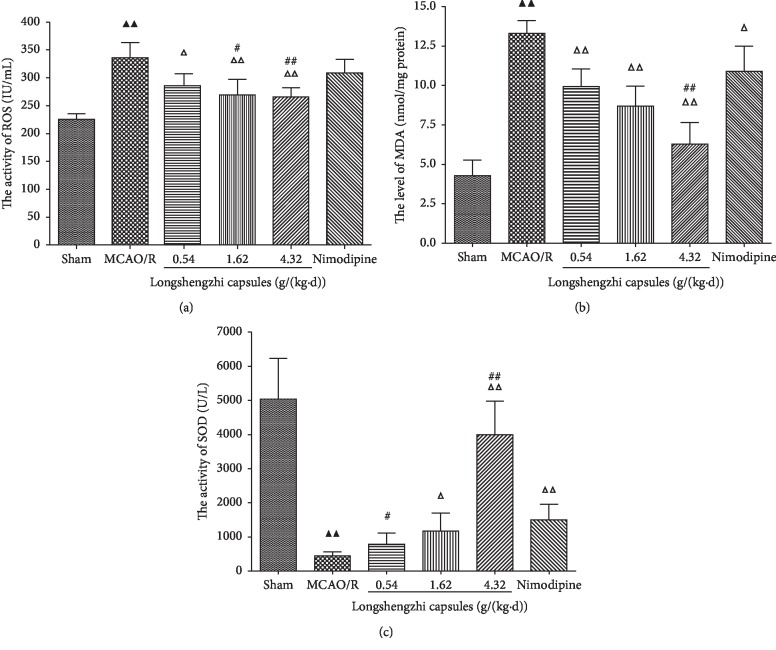
Effects of LSZ on ROS, MDA, and SOD levels for treatment of ischemic stroke in rats following MCAO/R. (a) Treatment with LSZ (0.54, 1.62, and 4.32 g/(kg·d)) significantly decreased ROS activity (b) and MDA concentration (c) while it increased SOD activity when compared with the MCAO/R group. Data are expressed as mean ± SD, *n* = 6 per group. ^▲^*P* < 0.05, ^▲▲^*P* < 0.01 vs sham group. ^Δ^*P* < 0.05, ^ΔΔ^*P* < 0.01 vs MCAO/R group. ^#^*P* < 0.05, ^##^*P* < 0.01 vs Nimodipine group.

In order to study the antioxidation effects of LSZ on focal embolic stroke in rats after MCAO/R, the expression of ROS, MDA, and SOD were measured in the ischemic penumbra of the cerebral cortex after seven days of treatment using ELISA assay kits. The activity of SOD was significantly lower while the concentration of MDA and ROS were higher in the MCAO/R group compared with those in the sham group (*P* < 0.01, Figure 3). Treatment with both LSZ (0.54, 1.62, and 4.32 g/(kg·d)) and Nimodipine (0.01 g/(kg·d)) significantly increased SOD activity and decreased MDA and ROS levels (*P* < 0.05 or *P* < 0.01, Figure 3), while Nimodipine (0.01 g/(kg·d)) did not change ROS levels significantly. In parallel with Nimodipine (0.01 g/(kg·d)), it showed that LSZ (4.32 g/(kg·d)) significantly increased SOD activity and decreased MDA and ROS levels (*P* < 0.01, Figure 3).

### 3.4. Effects of Longshengzhi Capsules on TNF-*α* and IL-1*β* Levels for Treatment of Ischemic Stroke in Rats following MCAO/R

**Figure 4:**
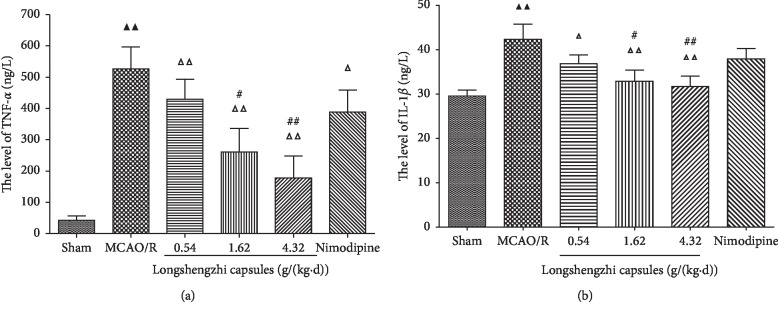
Effects of LSZ TNF-*α* and IL-1*β* levels for treatment of ischemic stroke in rats following MCAO/R. (a) LSZ treatment (0.54, 1.62, 4.32 g/(kg·d)) significantly decreased TNF-*α* (b) and IL-1*β* concentration when compared with the MCAO/R group. Data are expressed as mean ± SD, *n* = 6 per group. ^▲^*P* < 0.05, ^▲▲^*P* < 0.01 vs sham group. ^Δ^*P* < 0.05, ^ΔΔ^*P* < 0.01 vs MCAO/R group. ^#^*P* < 0.05, ^##^*P* < 0.01 vs Nimodipine group.

In order to figure out whether LSZ treatment could suppress the production of inflammatory mediators, this study focused on the expression of two proinflammatory mediators in the ischemic penumbra using ELISA assay kits. The MCAO/R group showed a higher expression of TNF-*α* and IL-1*β* than the sham group (*P* < 0.01, Figure 4). In parallel with the MCAO/R group, treatment with LSZ (0.54, 1.62, and 4.32 g/(kg·d)) and Nimodipine (0.01 g/(kg·d)) significantly reduced concentrations of TNF-*α* and IL-1*β* in the ischemic penumbra (*P* < 0.01or *P* < 0.05, Figure 4). However, no significant difference was found between Nimodipine group (0.01 g/(kg·d)) and MCAO/R group at the level of IL-1*β* (*P* > 0.05, Figure 4). In parallel with Nimodipine (0.01 g/(kg·d)), it showed that LSZ (1.62, 4.32 g/(kg·d)) significantly decreased TNF-*α* and IL-1*β* levels (*P* < 0.01 or *P* < 0.05, Figure 4).

### 3.5. Effects of Longshengzhi Capsules on Relative Levels of NF-*κ*B and VEGF for Treatment of Ischemic Stroke in Rats following MCAO/R

**Figure 5:**
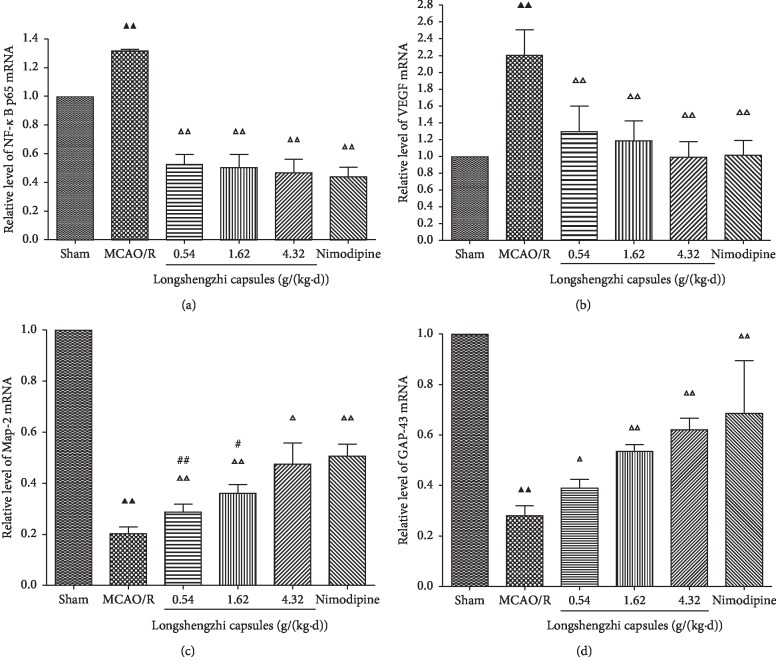
Effects of LSZ on levels of NF-*κ*B, VEGF, Map-2, and GAP-43 for treatment of ischemic stroke in rats following MCAO/R. (a) Treatment using LSZ (0.54, 1.62, and 4.32 g/(kg·d)) significantly decreased the relative levels of NF-*κ*B (b) and VEGF (c) and significantly increased the relative levels of Map-2 (d) and GAP-43 when compared with the MCAO/R group. Data are expressed as mean ± SD, *n* = 6 per group. ^▲^*P* < 0.05, ^▲▲^*P* < 0.01 vs sham group. ^Δ^*P* < 0.05, ^ΔΔ^*P* < 0.01 vs MCAO/R group. ^#^*P* < 0.05, ^##^*P* < 0.01 vs Nimodipine group.

After 1.5-hour ischemia, there were obvious differences in the relative levels of NF-*κ*B and VEGF in the MCAO/R group when compared with the sham group. NF-*κ*B, an inflammatory transcription factor, has been widely studied in stroke models. In order to explore the interaction of NF-*κ*B and VEGF in inflammatory and BBB damage, treatment with LSZ (0.54, 1.62, and 4.32 g/(kg·d)) and Nimodipine (0.01 g/(kg·d)) dramatically reduced the relative levels of NF-*κ*B and VEGF (*P* < 0.01, Figures 5(a) and 5(b)), suggesting that LSZ (0.54, 1.62, and 4.32 g/(kg·d)) has the capacity to protect rats that had been subjected to MCAO/R from inflammatory and BBB damage. In addition, there was no difference in decreasing relative levels of NF-*κ*B and VEGF between LSZ (0.54, 1.62, and 4.32 g/(kg·d)) and Nimodipine (0.01 g/(kg·d)) (*P* > 0.05, Figures 5(a) and 5(b)).

### 3.6. Effects of Longshengzhi Capsules on Relative Levels of Map-2 and GAP-43 for Treatment of Ischemic Stroke in Rats following MCAO/R

Map-2, a postsynaptic protein, and GAP-43, a presynaptic protein, are both involved in synaptogenesis. After 1.5-hour ischemia, there were clear differences in the relative levels of Map-2 and GAP-43 in the MCAO/R group when compared with the sham group. In order to assess neuroprotective effects, treatment with LSZ (0.54, 1.62, and 4.32 g/(kg·d)) and Nimodipine (0.01 g/(kg·d)) dramatically increased the relative levels of Map-2 and GAP-43 (*P* < 0.01 or *P* < 0.05, Figures 5(c) and 5(d)), which suggested that LSZ (0.54, 1.62, and 4.32 g/(kg·d)) promotes neurorestoration in rats that have been subjected to MCAO/R. Furthermore, LSZ (4.32 g/(kg·d)) and Nimodipine (0.01 g/(kg·d)) had same degree of effect on neurorestoration (*P* > 0.05, Figures 5(c) and 5(d)).

### 3.7. Effects of Longshengzhi Capsules on Relative Expression Levels of HIF-1*α*, MMP-2, MMP-9, p38 MAPK, and p-p38 MAPK for Treatment of Ischemic Stroke in Rats following MCAO/R

**Figure 6:**
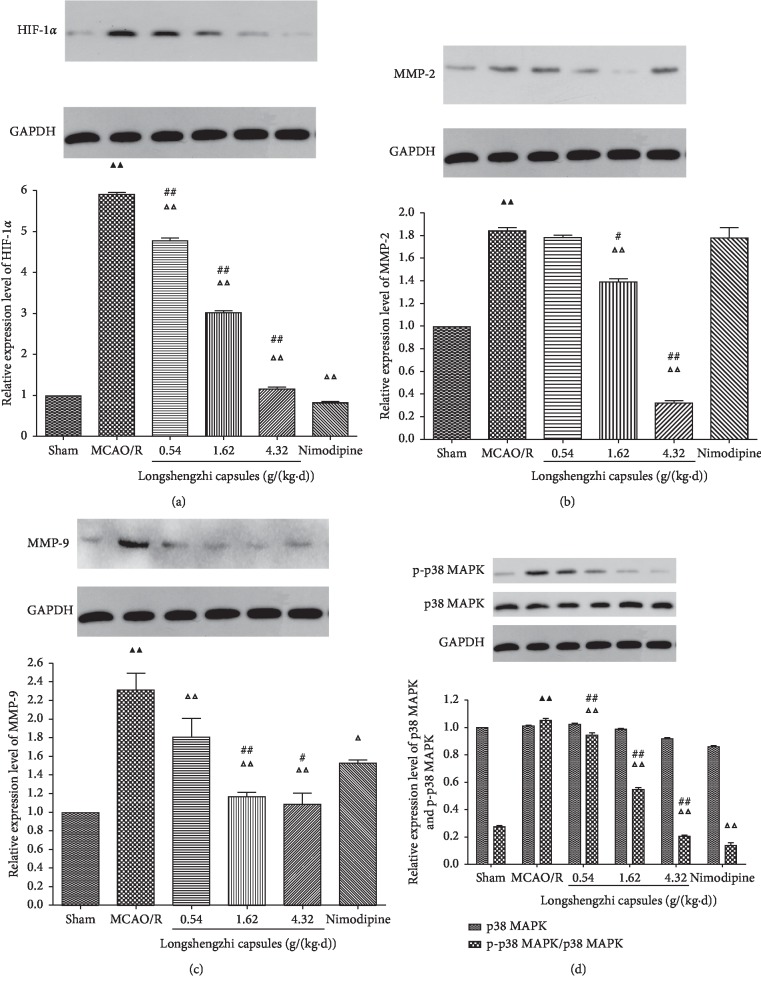
Effects of LSZ on relative expression levels of HIF-1*α*, MMP-2, MMP-9, p38 MAPK, and p-p38 MAPK for treatment of ischemic stroke in rats following MCAO/R. (a)–(d) Representative western blots of HIF-1*α*, MMP-2, MMP-9, GAPDH, p38 MAPK, and p-p38 MAPK. Treatment using LSZ (0.54, 1.62, and 4.32 g/(kg·d)) significantly decreased the relative levels of (a) HIF-1*α*, (b) MMP-2, (c) MMP-9 (d), and p-p38 MAPK/p38 MAPK when compared with the MCAO/R group. However, LSZ (0.54 g/(kg·d)) demonstrated no differences with the MCAO/R group on the relative expression level of MMP-2. Data are expressed as mean ± SD, *n* = 6 per group. ^▲^*P* < 0.05, ^▲▲^*P* < 0.01 vs sham group. ^Δ^*P* < 0.05, ^ΔΔ^*P* < 0.01 vs MCAO/R group. ^#^*P* < 0.05, ^##^*P* < 0.01 vs Nimodipine group.

In order to further explore the mechanisms of anti-inflammatory and neuroprotective effects, western blotting was conducted to assess the relative expression levels of HIF-1*α*, MMP-2, MMP-9, p38 MAPK, and p-p38 MAPK. In parallel with the sham group, all factors were dramatically upregulated following cerebral ischemia. In contrast, relative expression levels of p38 MAPK remained essentially stable in all groups. When compared with the MCAO/R group, treatment using LSZ (0.54, 1.62, and 4.32 g/(kg·d)) and Nimodipine (0.01 g/(kg·d)) significantly inhibited MCAO/R-induced upregulation for all factors (*P* < 0.01 or *P* < 0.05, Figure 6), whereas LSZ (0.54 g/(kg·d)) and Nimodipine (0.01 g/(kg·d)) had no obvious effect on relative expression levels of MMP-2 (*P* > 0.05, Figure 6(b)). In parallel with Nimodipine (0.01 g/(kg·d)), treatment with LSZ (1.62, 4.32 g/(kg·d)) significantly decreased the relative expression levels of HIF-1*α*, MMP-2, and MMP-9 (*P* < 0.01 or *P* < 0.05, Figures 6(a)–6(c)). However, Nimodipine (0.01 g/(kg·d)) had a better effect on reducing p-p38 MAPK than LSZ (1.62, 4.32 g/(kg·d)) (*P* < 0.01, Figure 6(d)).

## 4. Discussion

Reperfusion is the optimal choice for limiting brain injury after stroke, whereas restoration of blood flow is tightly associated with a sharp exacerbation of neural tissue injury and a profound inflammatory response known as reperfusion injury. In recent decades, drug combinations have been frequently investigated as researchers struggle to resolve this contradiction. TCM serves as a drug combination and focuses on multitarget therapy to treat various neurological diseases including stroke. A growing body of clinical evidence has established that LSZ fight against the recovery stages of cerebrovascular diseases and has performed well in improving neurological function [25–29, 36]. However, till now, its mechanism has not been clarified, which has severely hindered its clinical application and development. As a consequence, this study was devoted to two dominant research areas. On one hand, we focused on validating the anti-inflammatory and neuroprotective effects of LSZ. On the other hand, we set out to uncover the ways in which LSZ promote anti-inflammatory and neuroprotective effects.

The present study demonstrated that LSZ served as a blueprint for the ways in which traditional Chinese medicine could fight cerebral I/R injury in the MCAO/R model. These effects might be closely related to anti-inflammatory and neuroprotective pathways. Data around TTC staining, infarction volume, neurologic scores, and brain edema demonstrated that LSZ decreased the damage after MCAO/R when compared with the MCAO/R group. Notably, high doses of LSZ possessed optimal cerebral protective effects.

Inflammation is one of the main pathogenetic factors in ischemic stroke [47]. Ischemia and reperfusion injury triggered both the production and secretion of inflammatory cytokines and proinflammatory cytokines including TNF-*α* and IL-1*β* in rats. The upregulation of TNF-*α* and IL-1*β* in the MCAO/R group was hugely alleviated when rats were treated with LSZ, especially at the highest dose. In parallel with the positive drug (Nimodipine), LSZ had a better effect on the treatment of ischemic stroke. NF-*κ*B is commonly associated with the initiation of inflammatory responses [48]. Its properties have been shown to be extensively implicated in the immune system and rapid post-translational activation in response to various pathogenic signals in particular. NF-*κ*B possesses direct potential for participating in cytoplasmic/nuclear signaling, as well as to activate transcription of a variety of genes which encode immunologically relevant proteins [13]. Taken together, the NF-*κ*B signaling pathway is crucial for the regulation of inflammation following ischemic stroke. It means that suppression of the NF-*κ*B signaling pathway could downregulate inflammation and alleviate I/R injury [49–51]. This study has shown that LSZ contribute to the inhibition of proinflammatory cascades following ischemic stroke by suppressing the NF-*κ*B signaling pathway.

Oxidative stress is another crucial pathological factor in I/R injury. It has been robustly demonstrated that cerebral ischemia increases the level of reactive oxygen species while decreasing the activity of antioxidant enzymes in the cerebral cortex [52]. This study has shown that treatment with LSZ significantly increased SOD activity and decreased MDA levels in the ischemic penumbra of the cerebral cortex during the ischemia recovery phase. Oxidative stress and inflammation can induce each other and result in aggravated nerve injury [53]. Thus, this study has provided evidences for the potency of LSZ to fight against oxidant stress and inflammation.

BBB damage is a promising target for clinical intervention in ischemic stroke [54]. In the acute phase, ischemic damage results in a rapid activation of resident microglia in the brain. Microglial morphology changes from a ramified shape to an amoeboid shape [55, 56]. Reactive microglia/macrophages can be observed as early as two hours after an ischemic stroke and maintained for up to one week [57]. The production of proinflammatory mediators from microglia and astrocytes rapidly increases adhesion molecule expression on the endothelium. Following activation of peripheral leukocytes and infiltration into the brain, the tight junctions between endothelial cells of the BBB are disrupted and become more permeable [58–60]. In contrast, reactive microglia, platelets, and infiltrating leukocytes further release IL-1*β*, TNF-*α*, ROS, and MMPs that aggravate ischemic injury in MCAO [57, 61]. HIF-1*α* and its downstream VEGF have been demonstrated to play significant parts in BBB integrity following ischemic stroke [62]. The function of the VEGF is controversial in ischemic stroke. Thus far, the most compelling argument has been that VEGF increases BBB permeability in the early phase of ischemic stroke and subsequently facilitates neurovascular remodeling [63]. Further to this, MMPs have been shown to disrupt BBB integrity by altering tight junction proteins under ischemic and inflammatory conditions during the early phase of ischemic stroke [64, 65].

Consistent with a previous study which found that HIF-1*α* was implicated in the regulation of MMPs [66], this study has provided evidence to suggest that MMP-2/9 increased in line with the upregulation of HIF-1*α*. When taken together, we can see that BBB damage is attenuated via inhibition of HIF-1*α* with MMPs and VEGF. In an effort to clarify the neuroprotective mechanism of LSZ treatment, the expression level of HIF-1*α*, MMP-2/9, and VEGF were measured after seven days of treatment using LSZ. The data demonstrated that ischemia damaged BBB integrity, and MMP-2/9 upregulation was visible in the MCAO/R group. Treatment with LSZ significantly alleviated BBB damage induced by ischemic stroke, which was accompanied by the downregulation of MMP-2/9, VEGF, and HIF-1*α*. Further to this, previous evidence has shown that the upregulation of Map-2 and GAP-43 promoted dendrite branching and synaptogenesis [67]. Similarly, this study has suggested that LSZ possesses neural restoration effects.

Neuronal damage after ischemic stroke occurs through oxidative stress, inflammation, and BBB damage, leading to an apoptotic cascade. Recently, the significance of MAPK signaling pathways as both targets and mediators of cerebral ischemic reperfusion injury has been gradually recognized. Accumulating evidence has demonstrated that MAPK signaling pathways are involved in neuroprotective effects against I/R injury in mild transient focal cerebral ischemia [68, 69]. This study focused on p38 MAPK signaling as a means to uncover the anti-inflammatory mechanism of treatment with LSZ. p38 MAPK signaling is involved in reactive astrogliosis and plays a key role in the synthesis of proinflammatory mediators in the cortical penumbra [70]. These results suggested that LSZ could efficiently suppress p38 MAPK activation, whereas no effect on the expression level of p38 MAPK was found. Furthermore, some evidence has pointed to the fact that the p38 MAPK-NF-*κ*B p65 signaling pathway plays a role in regulating apoptosis in ischemic reperfusion injury [37]. Similarly, this study demonstrated that LSZ efficiently improved ischemic stroke along with the inhibition of p-p38 MAPK and NF-*κ*B. It appeared that LSZ played an anti-inflammatory role via p38 MAPK signaling pathway. However, further researcher is needed to confirm this hypothesis.

Overall, this study has provided comprehensive evidence that supports the potential effects of LSZ on anti-inflammatory and neuroprotective effects as well as their underlying mechanisms. LSZ has the potential to ameliorate neurological effects after ischemic stroke. Moreover, LSZ could downregulate the p38 MAPK and HIF-1*α*/VEGF signaling pathway in order to improve ischemic reperfusion injury in vitro. Clinical trials have demonstrated that the anti-ischemic stroke effect of LSZ might benefit in the improvement of the cerebral microcirculation and anti-inflammation [25–28]. This means that LSZ might function as a multitarget drug in the treatment of ischemic strokes.

## 5. Conclusion

In summary, this study has shown that the anti-inflammatory and neuroprotective effects of LSZ were closely related to attenuation of ROS, MDA, TNF-*α*, IL-1*β*, MMP-2/, and NF-*κ*B, as well as the upregulation of Map-2, SOD, and GAP-43. While further research is needed to uncover the significance of p38 MAPK and HIF-1*α*/VEGF signaling pathways in anti-inflammatory and neuroprotective effects, this study may represent a novel mechanism of LSZ in ischemic stroke. The study provided a new direction and insight for the study of LSZ.

## Figures and Tables

**Table 1:** All ingredients of LSZ.

Component latin name	Family	Local name	English name	Part used
Plants				
*Astragalus membranaeus (Fisch.) Bge.*	Leguminosae	Huang Qi	Milkvetch Root	Root
*Carthamustinctorius* L.	Compositae	Hong Hua	Carthamustinctorius	Flower
*Angelica sinensis (Oliv.) Diels*	Apiaceae	Dang Gui	*Angelica sinensis*	Root
*Ligusticum striatum DC.*	Apiaceae	Chuan Xiong	Ligusticumwallichii	Rhizome
*Prunus davidiana (CarriŠre) Franch.*	Rosaceae	Tao Ren	Peach Kernel	Dried seed
*Paeonia lactiflora Pall.*	Paeoniaceae	Chi Shao	Red Peony Root	Root
*Aucklandia lappa DC.*	Compositae	Mu Xiang	Costustoot	Root
*Acorus tatarionwii Schott.*	Araceae	Shi Chang Pu	Acori Tatarinowii Rhizoma	Root
*Taxillus balansae (Lecomte) Danser*	Loranthaceae	Sang Ji Sheng	Talxilli Herba	Stem
*Acanthopanax senticosus (Rupr. et Maxim.) Harms*	Araliaceae	Ci Wu Jia	Siberian Ginseng	Stem

Insects				
*Hirudo niponica Whitman*	Hirudinidae	Shui Zhi	Hirudo	Dried body
*Pheretima aspergillum (E. Perrier)*	Megascolecidae	Di Long	Pheretima	Dried body

**Table 2:** Oligonucleotide PCR primers.

Gene	Primer sequences (5′ to 3′)	Orientation
Rat GAPDH	GAAGGTCGGTGTGAACGGATTTG	Forward
	CATGTAGACCATGTAGTTGAGGTCA	Reverse
Rat NF-*κ*B p65	GCGAGACCTGGAGCAAGCCATTA	Forward
	GAGGCGGACCGCATTCAAGTCAT	Reverse
Rat VEGF	GTCACCACCACACCACCATCGT	Forward
	CTCCTCTCCCTTCATGTCAGGCT	Reverse
Rat GAP43	GGAGCCTAAACAAGCCGATGTG	Forward
	GGGTCTTCTTTACCCTCATCCTG	Reverse
Rat Map2	CTTGGTGCCCAGTGAGAAGAAAGT	Forward
	GCTGGTATTTGATGTTGTCGGTTG	Reverse

## Data Availability

The data used to support the findings of the study are included within the article.

## Abbreviations

MCAO/R: Middle cerebral artery occlusion and refusion
I/R: Ischemia and refusion
LSZ: Longshengzhi capsules
SOD: Superoxide dismutase
MDA: Malondialdehyde
ROS: Reactive oxygen species
TNF-*α*: Tumor necrosis factor *α*
IL-l*β*: Interleukin l*β*
r-tPA: Recombinant tissue plasminogen activator
FDA: Federal Drug Administration
TCM: Traditional Chinese medicine
BBB: Blood-brain barrier
MMPs: Matrix metalloproteinases
MAPKs: Mitogen-activated protein kinases
NF-*κ*B: Nuclear factor kappa-B
VEGF: Vascular endothelial growth factor
MMP-9: Matrix metalloproteinase-9
MMP-2: Matrix metalloproteinase-2
GAP-43: Growth-associated protein-43
MAP-2: Microtubule-associated protein-2
RT-PCR: Reverse transcription-polymerase chain reaction
HIF-1*α*: Hypoxia inducible factor-1*α*
TTC: 2,3,5-triphenyltetrazolium chloride
GAPDH: Glyceraldehyde-3-phosphate dehydrogenase.
